# Visual outcomes of Visian ICL implantation for high myopia in patients with shallow anterior chamber depth

**DOI:** 10.1186/s12886-019-1132-z

**Published:** 2019-05-29

**Authors:** Lingling Niu, Huamao Miao, Tian Han, Lan Ding, Xiaoying Wang, Xingtao Zhou

**Affiliations:** 1 Department of Ophthalmology and Optometry, Eye and ENT Hospital, Fudan University, 83 Fen Yang Road, Shanghai, 200031 China; 2 Key NHC Key Laboratory of Myopia (Fudan University), 83 Fen Yang Road, Shanghai, 200031 China; 3 Laboratory of Myopia, Chinese Academy of Medical Sciences, 83 Fen Yang Road, Shanghai, 200031 China; 4 Shanghai Research Center of Ophthalmology and Optometry, 83 Fen Yang Road, Shanghai, 200031 China

**Keywords:** High myopia, ICL V4c, Anterior chamber depth

## Abstract

**Background:**

High myopia with shallow anterior chamber depth (ACD less than 2.8 mm) is not rare. This observational study aims to evaluate visual outcomes after implantation of the Visian Implantable Collamer Lens with a central hole (ICL V4c) in these patients.

**Methods:**

A prospective cohort of consecutive 51 eyes of 31 patients (20 to 42 years old) was followed for at least 12 months (average 15.35 ± 4.90 months, rangers from 12 to 25 months). The preoperative ACD was 2.74 ± 0.04 mm (2.65 to 2.79 mm). Uncorrected distance visual acuity (UDVA), corrected distance visual acuity (CDVA), intraocular pressure (IOP), manifest refraction, vault, and endothelial cell density (ECD) were measured during the follow-ups after surgery.

**Results:**

All surgeries were performed safely and no complication was observed during the follow-ups. At the last follow-up, the safety index (postoperative CDVA / preoperative CDVA) was 1.33 ± 0.60 and the efficacy index (postoperative UDVA / preoperative CDVA) was 1.14 ± 0.54. After the surgery, no eye had decreased CDVA and 59% (30 eyes) of the eyes gained at least one line. Forty-seven eyes (92%) were within ±1.0 D and 35 eyes (69%) were within ±0.5 D of the attempted refraction. The mean postoperative vault was 380.00 ± 152.84 μm (90 to 700 μm). The ECD was reduced by 8.38 ± 0.06% as compared to the preoperative value (*p* < 0.001). No significant change was observed in IOP (*p* = 0.061) at the last follow-up. Ultrasound Biomicroscopy (UBM) showed none of the eyes had trabecular-iris angle closed.

**Conclusions:**

In this prospective observational study, ICL V4c implantation in patients with high myopia and shallow ACD achieved satisfying and stable visual outcomes. Its long-term safety and stability require further investigation.

**Trial registration:**

This trial was retrospectively registered on 05/08/2018 under the number (ChiCTR1800017594).

## Background

Since approved by the FDA in 2005, implantation of Implantable Collamer Lens (ICL™, STAAR Surgical, Nidau, Switzerland), a posterior chamber phakic intraocular lens, has been demonstrated a safe and effective way to correct high myopia [1, 2]. Advantages of ICL implantation include faster visual recovery, more stable refraction, and better visual quality than corneal refractive surgery [3–5]. However, an anterior chamber depth (ACD) no less than 2.80 mm is usually recommended [6] and patients with shallow ACD may be at a higher risk of implantation failure [7].

The CentraFLOW technology applied in the Visian ICL (ICL V4c), which is characterized by a 360 μm central hole that promotes natural circulation of the aqueous humor, can reduce the influence of metabolism on its own crystal, and alleviates the pain and discomfort caused by preoperative laser iridotomy [8, 9], resulting in decreased risks of cataracts, high intraocular pressure (IOP) and endothelial cell loss after ICL implantation [8–10]. However, it remains unknown whether implantation of ICL with a central hole is feasible in patients with shallow ACD (< 2.8 mm), a condition that is not rare in East Asian patients [11].

In this study we aimed to investigate the efficacy, safety, predictability and stability of ICL V4c implantation in patients with shallow ACD.

## Methods

### Subjects

A prospective, observational cohort study was carried out on consecutive patients who underwent ICL V4c implantation in the Department of Eye & ENT Hospital of Fudan University from April 2015 to May 2017. The study is adhered to the Declaration of Helsinki and was approved by the Ethics Committee of the Eye & ENT Hospital, Fudan University, China. All patients provided signed informed consent after a detailed explanation of risks and potential outcomes of the implantation and the study.

The baseline characteristics and preoperative biometric values of the patients are shown in Table Table 1. The study enrolled 51 eyes of 31 patients (4 men and 27 women, 36 ICLs and 15 Toric ICLs) with a mean age of 32.45 ± 6.85 years old. All patients underwent routine preoperative examinations and met the surgical indications of ICL implantation for the correction of high myopia. The inclusion criteria were: age between 20 and 45 years old, spherical refraction of over − 6.00 D, astigmatism of up to − 5.00 D, corrected distance visual acuity (CDVA) of 20/200 or better, stable refractive error (≤0.50 D of refractive error change in the past 2 years), no contact lens use for 2 weeks, and ACD < 2.80 mm. The exclusion criteria includes a history of ocular conditions other than myopia with or without astigmatism (suspicion of keratectasia, cornea or lens opacity, retinal detachment, glaucoma, macular degeneration, or neuro-ophthalmic disease), a history of inflammation or trauma, any chronic systemic disease, ocular surgery, and an endothelial cell count < 2000 cells/mm^2^.

**Table 1:** Preoperative Patient Demographic Data in Eyes Undergoing Implantation of ICL V4c

Characteristics	Mean ± SD (*N* = 51 eyes)	Range (Minimum, Maximum)
Age (y)	32.45 ± 6.85	20, 42
Follow-up (moths)	15.35 ± 4.90	12, 25
CDVA (Decimal)	0.86 ± 0.31	0.1, 1.2
Spherical refractive error (D)	−13.31 ± 4.34	−6.50, − 25.50
Cylindrical refractive error (D)	−1.50 ± 1.01	−4.00, 0
Spherical equivalent (D)	− 14.03 ± 4.46	− 7.50, − 25.75
ACD (mm)	2.74 ± 0.04	2.65, 2.79
WTW (mm)	11.67 ± 0.33	11.0, 12.9
IOP (mmHg)	15.76 ± 2.68	9.7, 20.9
ECD (cells/mm^2^)	3235.08 ± 478.07	2379, 4132
Axial length(mm)	28.30 ± 2.01	24.18, 32.84

*CDVA* = Corrected distance visual acuity; *D* = Diopter; *ACD* = Anterior chamber depth; *WTW* = White-to-white; *IOP* = Intraocular pressure; *ECD* = Endothelial cell count

Data are mean ± SD unless otherwise indicated

### ICL V4c

The Visian Implantable Collamer Lens with a 360 μm central hole (ICL V4c, STAAR Surgical, Nidau, Switzerland) is made from collamer, a biocompatible hydrophilic copolymer and hydroxyethyl methacrylate with an ultraviolet light-filtering chromophore [2]. The implantable lens were used for correction of − 0.50 D to − 18.00 D myopic spherical refraction and up to − 5.00 D cylindrical refraction. The diameter (12.1 mm, 12.6 mm, 13.2 mm, and 13.7 mm) of ICL V4c was individually selected based on the horizontal white-to-white (WTW) distance and the ACD *as per* the manufacturer’s recommendations. Power calculation of the ICL V4c was performed using the software provided by the manufacturer and a modified vertex formula.

### Surgical technique and follow-ups

All implantations were performed by two experienced surgeons (XZ and XW). The surgical technique has been previously described by Chen X et al. [12]. Briefly, pupils were dilated prior to ICL V4c implantation. After injection of 1% sodium hyaluronate into the anterior chamber, an ICL V4c was implanted via a 3.0 mm clear corneal incision using an injector cartridge. It was then placed in the posterior chamber. Afterwards, the viscoelastic surgical agent was washed away using a balanced salt solution, and a miotic agent was instilled. After the surgery, patients were given 1% tobramycin dexamethasone for 3 days followed by 0.1% fluorometholone (tapered gradually over 2 weeks), 0.5% left ofloxacin for 1 week, non-steroidal anti-inflammatory (NSAID) eye drops for 2 weeks, and artificial tears for 1 month.

Patients were then followed at 1 day, 1 week, 1 month, 6 months, 12 months and 24 months after the surgery. All the patients were followed for at least 12 months. The mean follow-up time was 15.35 ± 4.90 months (ranges from 12 to 25 months). Routine measurements before and after the surgery include: decimal of uncorrected distance visual acuity (UDVA), decimal of corrected distance visual acuity (CDVA), manifest refraction (spherical equivalent, SE), intraocular pressure (IOP; non-contact tonometer, Canon, Japan), endothelial cell density (ECD; noncontact specular microscopy, SP-2000P, Topcon Corporation, Japan), axial length (IOL master, Carl Zeiss, Germany), anterior chamber depth (ACD; Pentacam, Oculus, Germany; measured from the corneal endothelium to the anterior lens), standard slit-lamp biomicroscopic and funduscopic examinations, central corneal thickness (Pentacam), horizontal corneal diameter (white-to-white, WTW; IOL master) and ultrasound biomicroscopy (UBM; Quantel medical, France).

### Statistical analysis

All statistical analyses were performed using SPSS Version 20.0 (SPSS, Chicago, IL, USA). The results were expressed as the mean ± standard deviation (SD). The Kolmogorov–Smirnov test was used to determine if a variable is normally distributed. The paired *t* test was used for normally distributed data and the Wilcoxon signed-rank test for abnormal distributed data. A *p* value < 0.05 was considered statistically significant. Statistical analysis for visual acuity was based on Decimal units. Standardized graphs for refractive surgery results were plotted using Microsoft Excel according to the refractive outcomes at 1 month, 6 months, 12 months and 24 months in all the patients.

## Results

All procedures were completed successfully and no complication was observed during the follow-up periods. The mean preoperative ACD for all patients was 2.74 ± 0.04 mm (2.65 to 2.79 mm). The preoperative mean CDVA was 0.86 ± 0.31 (0.1 to 1.2) and the mean SE was − 14.03 ± 4.46 diopters (D) (− 7.50 to − 25.75 D). Twelve eyes had preoperative SE over − 18.00 D.

### Safety and efficacy

**Fig. 1:**
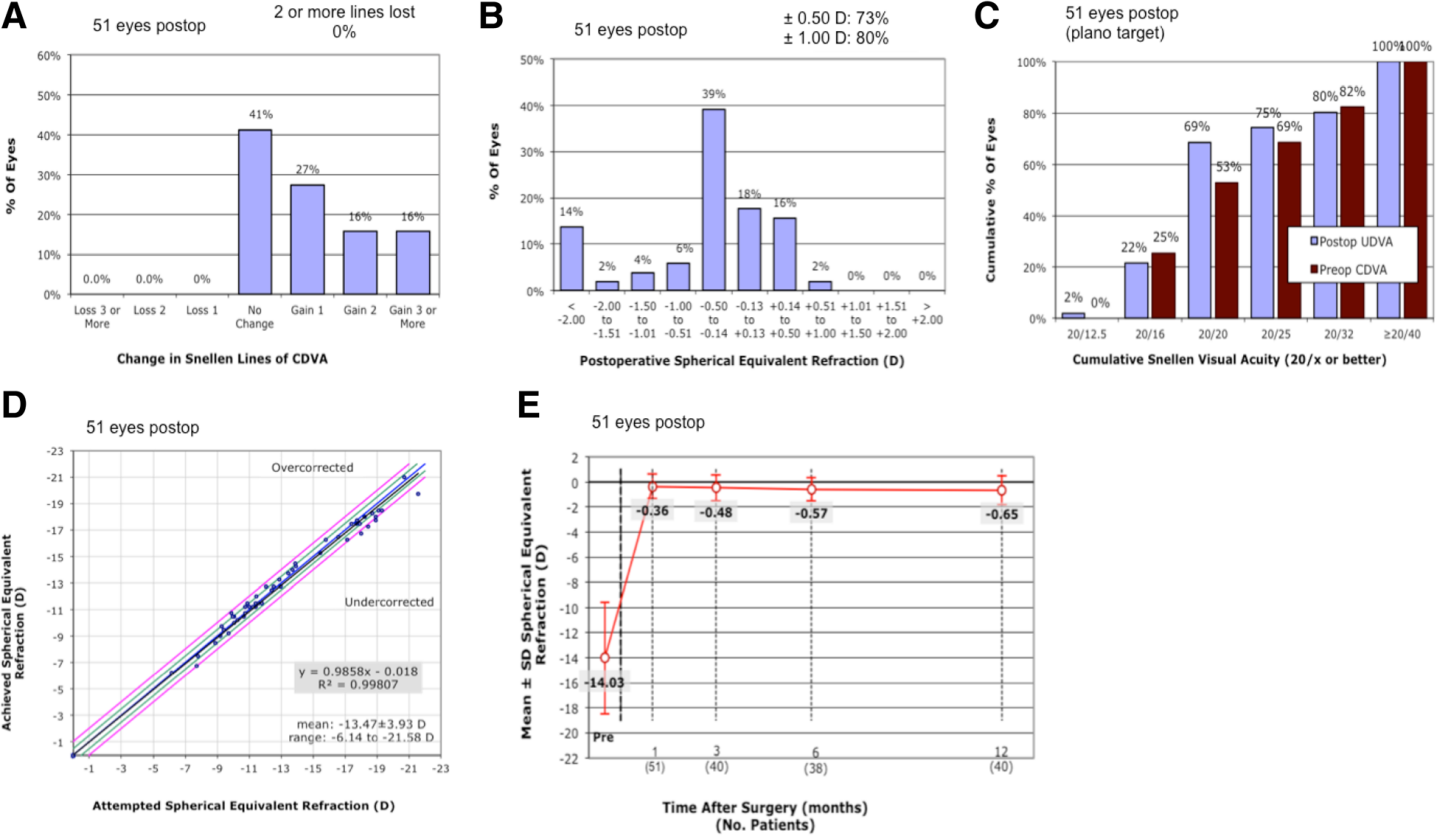
Clinical outcomes of 51 myopia eyes with shallow anterior chamber depth (ACD) after implantation of ICL with a central hole (ICL V4c). Changes of corrected distance visual acuity (CDVA) (**a**), distribution of postoperative spherical equivalent refraction (**b**), cumulative percentage of the eyes attaining specified cumulative levels of uncorrected distance visual acuity (UDVA) (**c**), attempted spherical equivalent refraction change versus the achieved spherical equivalent refraction change (**d**) at the last follow-up were plotted. Stability of postoperative spherical equivalent refraction was evaluated up to 12 months after surgery (**e**)

At the last follow-up, the mean postoperative CDVA was 1.00 ± 0.27 (0.4 to 1.5). The safety index (postoperative CDVA / preoperative CDVA) was 1.33 ± 0.60. No patient had CDVA loss at the final follow-up. Overall, 59% (30 eyes) of the patients had CDVA increased by at least 1 line over the preoperative CDVA. In 16% (8 eyes) of the eyes, CDVA increased by 3 lines or more. (Fig. 1a).

The mean postoperative UCVA was 0.89 ± 0.30 (0.12 to 1.2), and the efficacy index (postoperative UDVA / preoperative CDVA) was 1.14 ± 0.54 at the last follow-up. Thirty-seven eyes (73%) achieved a residual SE within ±0.5 D, and 41eyes (80%) achieved a residual SE within ±1.0 D (Fig. 1b). Among all the patients, 35 eyes (69%) had a postoperative UCVA of 20/20 or better (Fig. 1c). All 22 eyes with Toric ICL had a postoperative astigmatism of no more than 1.0 D.

### Predictability and stability

A scatter plot of the attempted versus achieved SE corrections is shown in Fig. 1d. Postoperatively, 35 eyes (69%) were within ±0.5 D of the attempted SE. Forty-seven eyes (92%) had a postoperative SE within ±1.0 D of the attempted SE. Additionally, all cases with a preoperative SE more than − 18.00 D (39 eyes) were within ±1.0 D of the attempted SE. The mean SE were − 0.36 ± 0.98 D and − 0.65 ± 1.15 D at 1 month (*n* = 51) and 12 months (*n* = 40) after surgery, respectively. The mean SE of 51 eyes at the last follow-up was − 0.67 ± 1.29 D, which was not significantly different from the result at 12 months (*p* = 0.457). (Fig. 1e) Four eyes had corrected SE exceeded ±1.0 D of the attempted SE at the last follow-up. Their refraction changes between 1 month and the last follow-up after surgery were over 1.0 D and their axial lengths increased by 0.10 to 0.31 mm as compared to the preoperative values.

### Intraocular pressure (IOP)

The preoperative trabecular-iris angle was 33.22° ± 3.72°(27.9° to 45.2°) and reduced to 19.03° ± 4.24°(11.0° to 27.1°) after surgery. No eye had a closed trabecular-iris angle as evaluated by UBM examinations. At the last follow-up, the average IOP was 15.15 ± 2.57 mmHg, which was not significantly different from the preoperative IOP (*p* = 0.061).

### Corneal endothelial cell density (ECD)

The postoperative corneal ECD was 2963.64 ± 396.17 cells/mm^2^ at the last follow up, which equals to an 8.38 ± 0.06% reduction to the preoperative ECD (*p* < 0.001). However, no eye decreased to less than 2000 cells/mm^2^ or had a significant ECD loss (≥ 30%) (Table 2). Moreover, no significant correlation was observed between the preoperative ACD and the ECD change at the last follow up (Pearson correlation coefficient *r* = 0.169, *p* = 0.286).

**Table 2:** Clinical Outcomes in Eyes Undergoing Implantation of ICL V4c At The Last Visit

Characteristics	Mean ± SD (51 eyes)	Range (Minimum, Maximum)
UDVA (Decimal)	0.89 ± 0.30	0.12, 1.2
CDVA (Decimal)	1.00 ± 0.27	0.4, 1.5
Spherical equivalent (D)	−0.67 ± 1.29	− 6.00, 0.75
Implanted ICL size (mm)	12.58 ± 0.31	12.1, 13.7
IOP (mmHg)	15.15 ± 2.57	9.7, 20.4
ECD (cells/mm^2^)	2963.64 ± 396.17	2358, 3651
Axial length(mm)	28.33 ± 2.15	24.19, 33.15
Vault (mm)	380.00 ± 152.84	90, 700

*CDVA* = Corrected distance visual acuity; *UDVA* = Uncorrected distance visual acuity; *IOP* = Intraocular pressure; *ECD* = Endothelial cell density; Data are mean ± SD unless otherwise indicated

### Vault

**Fig. 2:**
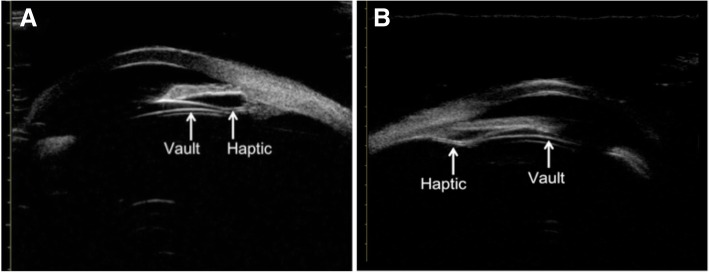
Locations of the ICL V4c in the eyes with vaults less than 200 μm at the last follow-ups. Images were captured by ultrasound biomicroscopy (UBM). The haptic of ICL V4c was on the ciliary processes in three eyes (**a**) and were at the posterior segment of the ciliary process in the forth eye (a representative image shown in **b**)

The average vault was 380.00 ± 152.84 μm (90 to 700 μm) at the last follow-up. Forty eyes (78.4%) had vault within 250 to 750 μm. In four eyes (7.8%) with vault less than 200 μm, three of them had haptics located on the ciliary processes (Fig. 2a) and the other had haptic located at the posterior segment of the ciliary processes as revealed by UBM examination (Fig. 2b). No obvious cataract was observed in these four eyes, and their UBM images showed no significant enhancement in crystal echo.

## Discussion

Since 2014, ICL V4c has been implanted in more than 60,000 eyes in the mainland of China, and the outcomes appeared to be satisfying [5, 12–14]. However, a recommended condition of ACD over 2.80 mm may restrict its use in patients with shallow ACD, a condition that is not rare in East Asian patients [11]. In this study we presented the short-term (12–25 months) clinical results after the implantation of ICL V4c for high myopia in patients with shallow ACD (< 2.8 mm).

No complication such as cataract was observed during and after the surgery in the studying cohort. Using ICL without a central hole, Lim reported subcapsular cataract in 2 out of 18 eyes with preoperative ACD of 2.71 ± 0.08 mm (ranges 2.42 to 2.79 mm) and the ICL were replaced for these two eyes [7]. The use of CentraFLOW technology in ICL V4c may improve the dynamic circulation of the aqueous humor and reduces the influence of metabolism on its own crystal [8]. In this study, the mean percentage of endothelial cell loss was 8.44%, and no eye had an ECD less than 2000 cells/mm^2^ or an ECD loss greater than 30%. These results are similar to the FDA-reported ECD loss of 8.9% at 3 years and 7.7% at 5 years after the surgery in patients with normal ACD. However, the follow-up time frame was different in our study [15, 16]*.* Moreover, in the current study, despite in the current study no significant association between the preoperative ACD and the ECD change at the last follow-up was observed, it has to be noted that the range of ACD was very narrow (2.65–2.79 mm) and the patient number was also limited. It is therefore hard for us to conclude that this result represents the true correlation between the two. To address this question, randomized trials with more patients and wider ACD ranges, including both shallow and normal ACD, should be carried out.

In all 51 eyes, 47 eyes (92%) were within ±1.0 D of the attempted SE, and 35 eyes (69%) were within ±0.5 D at the final follow-up, which is similar to the previous study in normal ACD patients by Sanders et al. showing that 88.2% of the patients were within ±1.0 D and 67.5% were within ±0.5 D of attempted correction at 3 years after the surgery [16]. In four eyes the achieved SE were more than ±1.0 D of the attempted SE and the refractive changes exceeded 1.0 D between 1 month after the surgery and the last follow-up. The preoperative manifest refraction values of these eyes were over − 18.00 D. We found that in these patients, the axial length increased by 0.10 to 0.31 mm, resulting in mild myopia regression at the last follow-up. Kamiya [17] also found that axial length had increased more than 0.5 mm at 8 years after surgery in eyes with a preoperative axial length of more than 27.5 mm. Thus the elongation of axial length may affect the predictability and stability of ICL implantation in high myopia.

In our study, four eyes (7.8%) had a vault less than 200 mm at the last follow-up, in which haptics were not placed right in the ciliary sulcus. Although no obvious cataract occurred in these eyes and UBM showed no significant enhancement in crystal echo, whether low vault may cause complications such as anterior subcapsular opacification in these patients may need further investigation [18].

Narrowing of the trabecular-iris angle is another concern in patients with shallow ACD. Fernandez et al. reported that after ICL V4c implantation, the trabecular-iris angle was reduced by 34.5 to 42% at 3 months after surgery in patients with normal ACD [19], which is similar to our results (42.7% reduction after the surgery). Alfonso et al. reported that eyes with acute increases in intraocular pressure (IOP) were significantly more myopic and had shallower ACD [20]. However, no postoperative angle closure or abnormal IOP was observed during the follow-up in this study. The mean IOP was 15.15 ± 2.57 mmHg, which was not significantly different from the baseline. Nevertheless, considering the trabecular-iris angle narrows with increased age and crystalline lens height [21], the trabecular-iris angle and IOP should be closely followed for longer period of time.

This study has several limitations. Firstly, the number of patients was relatively small and the average follow-up time was only 15 months. Complications may be revealed in larger population and over longer follow-up period. Therefore, continuous surveillance in these patients is very important to further validate efficacy and safety of the procedure. Secondly, although the clinical data were prospectively collected, the results need to be further verified by randomly controlled studies as compared to patients with deeper ACD. Such studies may finally justify the feasibility of ICL V4c in patients with shallow ACD.

## Conclusions

In this prospective observational study, ICL V4c implantation in patients with ACD less than 2.8 mm was shown to be a safe, effective and stable way to correct high myopia. There was no significant endothelial loss or increase in IOP. Patients could achieve high, stable operative visual outcomes one year after the surgery.

## Abbreviations

ACD: Anterior chamber depth
CDVA: Corrected distance visual acuity
D: Diopter
ECD: Endothelial cell density
ICL: Implantable Collamer Lens
IOP: Intraocular pressure
NSAID: Non-steroidal anti-inflammatory drugs
SD: Standard deviation
SE: Spherical equivalent
UBM: Ultrasound Biomicroscopy
UDVA: Uncorrected distance visual acuity
WTW: White-to-white
